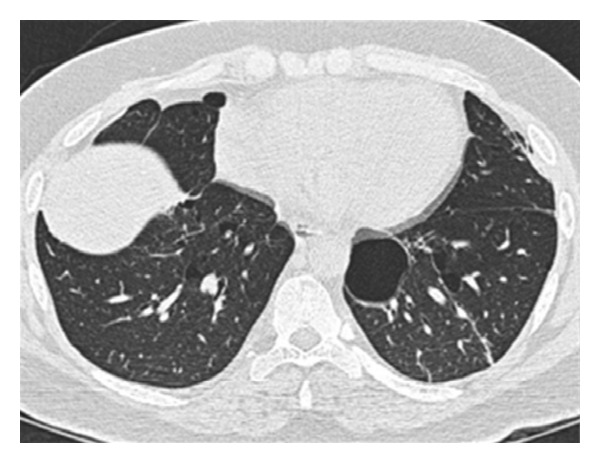# A Young Man with Bilateral Spontaneous Pneumothorax

**DOI:** 10.1155/2011/414165

**Published:** 2011-10-01

**Authors:** Liese Lieve Willemien Verhaert

**Affiliations:** Catharina Hospital, Eindhoven, The Netherlands

## Abstract

*Case.* A 33-year-old male nonsmoker presented with sudden onset of dyspnoea and thoracic pain. Chest radiograph showed a left-sided pneumothorax. Few days later he developed a right-sided pneumothorax. He had a positive family history of pneumothorax. High-resolution computed tomography of the chest showed multiple pulmonary cysts predominantly located in the lower lung regions. We suspected Birt-Hogg-Dubé syndrome (BHD). *Conclusion.* Birt-Hogg-Dubé syndrome is a rare autosomal dominant inherited genodermatosis and characterised by clinical manifestations including hamartomas of the skin, renal tumors, and pulmonary cysts with spontaneous pneumothorax. BHD is probably underdiagnosed because of the wide variability in its clinical expression. It is important to recognize these patients because of the possibility of developing renal cancer.

## 1. Case

**Figure 1:**
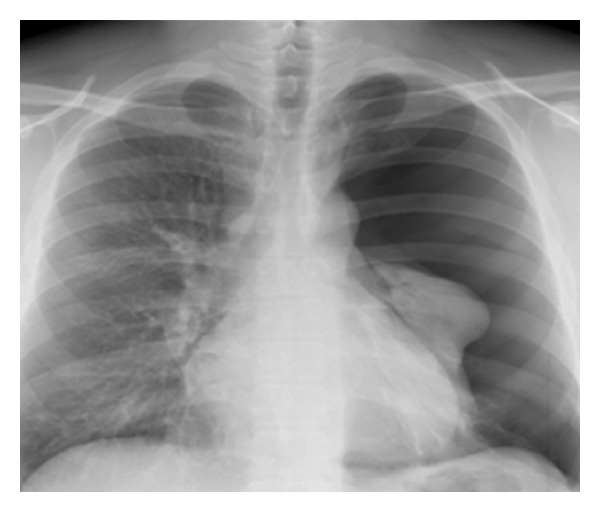


A 33-year-old male nonsmoker presented with sudden onset of dyspnoea and thoracic pain. He had normal haemodynamics and a peripheral blood oxygen saturation of 95% by clinical examination. He had no skin lesions. Chest radiograph showed a left-sided pneumothorax (Figure 1).

A detailed family history suggested an aunt who had also suffered a spontaneous pneumothorax.

Next day, patient underwent a thoracoscopy under local anaesthesia. There was seen a big bulla in the left lung top. For this reason, patient received a thoraxdrain, followed by surgical intervention with a bullectomy and pleurectomy.

Few days later, he had a right-sided pneumothorax. Next day, he received again surgical intervention on the right side with bullectomy and pleurectomy.

Further investigation showed normal alfa-1-antitrypsin in the lab analysis. Radiological investigation by high-resolution computed tomography of the chest after bullectomy showed multiple pulmonary cysts predominantly located in the lower lung regions (Figure 2). 

Because of bilateral pneumothoraces, positive family history, and pulmonary cysts, we suspected Birt-Hogg-Dubé syndrome (BHD). Genetic investigation showed indeed mutation in exon 10 (c.655dupG) of the FLCN gene what confirmed the diagnosis of BHD.

## 2. Syndrome

Familial spontaneous pneumothorax is rare. Approximately 10% of patients who have spontaneous pneumothorax have a positive family history [1–3]. 

Spontaneous pneumothoraces have been described in inherited disorders as alfa-1-antitrypsin deficiency, Marfans syndrome, tuberous sclerosis, cystic fibrosis, Ehlers-Danlos syndrome, and Birt-Hogg-Dubé syndrome. 

Birt-Hogg-Dubé syndrome is a rare autosomal dominant inherited genodermatosis and characterised by clinical manifestations including hamartomas of the skin (fibrofolliculomas, trichodiscomas, acrochordons, etc.), renal tumours, and pulmonary cysts with spontaneous pneumothorax. Patients with BHD syndrome do not always have all three manifestations. Patients can have unrecognised fibrofolliculomas or even no skin manifestations at all [3–5]. Patients with spontaneous pneumothoraces, skin signs, or BHD features in family members can lead the diagnosis. BHD-associated renal cancer shares general features with other types of hereditary renal tumours: early age at onset and multifocal or bilateral disease. Preventive measures are aimed mainly at early diagnosis and treatment of renal cancer. 

### 2.1. Genetics

In 1977, A. R. Birt, G. R. Hogg, and W. J. Dubé originally described a syndrome of multiple hamartomas of the hair follicle, higher incidence of renal neoplasia (sevenfold increased risk), and spontaneous pneumothorax (50-fold increased risk). All reported families present an autosomal dominant condition caused by germline mutations involving the Folliculin (FLCN) gene located on chromosome 17p11.2. The function of this protein is largely unknown, although FLCN has been linked to the mTOR pathway. Folliculin is highly expressed in a variety of tissues, including the skin, kidney, and lung (stromal cells and type I pneumocytes). Somatic second hit mutations identified in BHD-associated renal tumours are consistent with a tumour-suppression function for FLCN [1–5].

### 2.2. Clinical Manifestations

Skin lesions usually appear after the age of 20 years, as multiple, dome-shaped, whitish papules predominantly on the face, scalp, neck, and upper chest. These lesions are mainly on the nose and cheeks. Histologically, the skin tumours are benign hair follicle tumours designated as fibrofolliculoma. Therapeutic options are limited and often not curative (laser ablation, shave and cautery treatment, curettage, and excision).

The most threatening complication of BHD is renal cancer. 27% of patients with BHD develop renal cancer at a mean age of 50.4 years. 

Chromophobe renal cancer and a mixed pattern of chromophobe and oncocytic renal tumours are typical for patients with BHD [3, 6].

There are no current guidelines regarding surveillance of renal cancer. The main methods are CT, MRI, and renal ultrasonography. Annual renal MRI seems to be the best available surveillance method, with high sensitivity and no radiation side effects. 

### 2.3. Radiology

More than 80% of patients with BHD have multiple lung cysts on CT examination of the thorax. The lung cysts are multiple thin-walled cysts of various size, predominantly located in the lower medial and subpleural regions. The histology of cysts is consistent with emphysematous changes [2, 7].

Cystic lung disease seen in BHD syndrome needs to be distinguished from other lung diseases characterized by multifocal or diffuse cystic changes, including lymphangioleiomyomatosis, pulmonary Langerhans cell histiocytosis, lymphocytic interstitial pneumonitis, and Pneumocystis pneumonia. 

### 2.4. Pulmonary Function Tests

Small studies looked at the characteristics of patients with BHD. There were no specific lung function test results [2].

## 3. Conclusion

Birt-Hogg-Dubé syndrome is a rare autosomal dominant inherited genodermatosis and characterised by clinical manifestations including hamartomas of the skin, renal tumors, and pulmonary cysts with spontaneous pneumothorax.

BHD is probably underdiagnosed because of the wide variability in its clinical expression. Think about the diagnosis by a patient who presents with (bilateral) spontaneous pneumothorax and a positive family history of pneumothoraces.

It is important to recognize these patients because of the possibility of developing renal cancer. Annual renal MRI seems to be the best available surveillance method, with high sensitivity and no radiation side effects.

## Figures and Tables

**Figure 2:**